# Circulating oxylipins predict mortality in heart failure with preserved ejection fraction

**DOI:** 10.1093/eschf/xvaf010

**Published:** 2026-01-08

**Authors:** Zeeshan Ali, Muhammad Anwar, Wasiq Subhani, Syeda Alizay Kirmani

**Affiliations:** Department of Medicine, Liaquat College of Medicine & Dentistry, Bahria Town, Karachi 75500, Pakistan; Department of Internal Medicine, Bolan Medical College & Hospital, Quetta, Pakistan; Department of Medicine, Liaquat College of Medicine & Dentistry, Bahria Town, Karachi 75500, Pakistan; Department of Medicine, Liaquat College of Medicine & Dentistry, Bahria Town, Karachi 75500, Pakistan

**Keywords:** HFpEF, Heart failure with preserved ejection fraction, Oxylipins, Lipid mediators, Prognostic biomarkers, Mortality prediction

Dear Editor, We came across an insightful article by Aradhyula *et al*.^1^ titled ‘Circulating oxylipins predict mortality in heart failure with preserved ejection fraction’. The study provides an innovative perspective on lipid mediators as potential prognostic biomarkers in HFpEF, a syndrome still lacking robust diagnostic and therapeutic targets. While we commend the authors for addressing this complex area, several methodological and interpretative aspects merit clarification before these findings are translated into clinical practice.

The analysis was conducted on a relatively small cohort derived from a tertiary-care population, which may not accurately represent the heterogeneity of real-world HFpEF.^1^ Given the demographic and comorbidity diversity in HFpEF, particularly across sex, ethnicity, and metabolic phenotype,^2,3^ the external validity of the proposed oxylipin signature remains uncertain. Stratified analyses or validation in larger, multicentric registries could improve the robustness and generalizability of the results.^4^

The quantification of oxylipins by LC–MS/MS requires stringent pre-analytical standardization, as these lipid mediators are highly sensitive to temperature, oxidation, and sample handling.^5^ The article did not clearly describe storage conditions, internal standards, or batch-normalization procedures. Without these details, analytical reproducibility becomes difficult to ensure, particularly across laboratories. Reporting of intra- and inter-assay coefficients of variation and blinded duplicate analyses would strengthen confidence in the biomarker stability.^6^

While the multivariable Cox regression incorporated conventional predictors, residual confounding cannot be excluded. Key pathophysiological contributors such as renal function trajectories, inflammatory biomarkers, and medication effects (e.g. statins, ACE inhibitors, SGLT2 inhibitors) were not clearly addressed.^7^ Moreover, lipid metabolism can be profoundly influenced by nutritional status and comorbid diabetes.^8^ Adjustment for these variables, or sensitivity analyses excluding patients with advanced chronic kidney disease or diabetes, would clarify whether oxylipins represent independent prognostic factors or merely correlate with established risk determinants.

The temporal relationship between baseline oxylipin levels and subsequent mortality requires careful interpretation. As the median follow-up was relatively short, the study may reflect associations rather than causal pathways.^1^ Reverse causation is possible if elevated oxylipins result from acute decompensation or systemic inflammation rather than being antecedent markers.^9^ Longitudinal profiling with repeated sampling could help delineate whether oxylipins act as triggers, mediators, or epiphenomena of worsening HFpEF.

The exploratory multimetabolite modelling raises the potential for overfitting. Given the modest sample size relative to the number of metabolites evaluated, the event-per-variable ratio might have been insufficient.^10^ Although cross-validation was performed, an independent validation cohort would have provided stronger evidence for model stability. We encourage the authors to make their data and analytical code publicly available to facilitate external replication and meta-analytical synthesis.

In summary, Aradhyula *et al*. should be commended for exploring the lipidomic dimension of HFpEF prognosis. However, issues related to cohort representativeness, analytical standardization, confounding adjustment, and statistical power warrant cautious interpretation. Until confirmed in independent studies with harmonized methodologies, the clinical application of oxylipin profiling in HFpEF remains premature.
